# Transplant Coordinators' Perceived Impact of Availability of Multiple Generic Immunosuppression Therapies on Patients, Workload, and Posttransplant Maintenance Therapy

**DOI:** 10.1155/2013/897434

**Published:** 2013-01-08

**Authors:** K. Parker, E. A. Zagadailov, A. S. Bruno, A. M. Wiland

**Affiliations:** ^1^Division of Nephrology and Hypertension, Department of Internal Medicine, East Carolina University, Greenville, NC 27858, USA; ^2^Department of Global Health Economics and Outcomes Research, Xcenda, LLC, Palm Harbor, FL 34685, USA; ^3^Department of Medical Affairs, Novartis Pharmaceuticals Corporation, East Hanover NJ 07936, USA

## Abstract

*Background*. No studies have evaluated the impact of multiple generic immunosuppression medications on transplant coordinators (TCs) and patients. *Methods*. A cross-sectional, multicenter online survey of TCs managing transplant recipients' outpatient immunosuppression was undertaken to assess TCs' perceptions of the impact of multiple generic immunosuppression therapies on patients and workload. *Results*. Forty-six of 106 transplant centers contacted (43%) completed the survey, with usable information from 34 TCs (53% in centers performing >100 solid organ transplants annually, 82% registered nurses, and 68% with >5-year experience working with transplant patients). TCs indicated that “change in strength,” “switching from branded to generics,” “heavy pill burden,” and “switching from one generic to another” were the four most frequent reasons for patient confusion regarding immunosuppression. TCs reported increased patient confusion over the previous year for patients on generic immunosuppression therapy: 44% answered ≥3 patient calls/day regarding confusion over immunosuppression therapy. Most TCs indicated increased workload since the introduction of generic immunosuppression therapy. TCs perceived “acute rejection rates,” “rate of graft loss,” and “poor patient adherence” as the three most likely consequences of multiple generic immunosuppression therapy. *Conclusion*. TCs associated availability of multiple generic immunosuppression therapy with increased patient confusion and time spent addressing patient concerns.

## 1. Introduction

Immunosuppression regimens containing mycophenolate mofetil (MMF) and tacrolimus (TAC) have demonstrated effectiveness to prevent acute rejection in solid organ transplant recipients [1, 2]. Furthermore, MMF and TAC are listed as preferred agents in solid organ transplant clinical practice guidelines such as the Kidney Disease Improving Global Outcomes (KDIGO) [3, 4]. Since the approval of the first generic MMF and TAC in July 2008 and August of 2009, respectively, there have been 10 generic manufacturers of MMF and 4 generic manufacturers of TAC that have emerged [5]. The American Society of Transplantation (AST) supports the use of generic immunosuppressants in transplant patients but stresses the need for bioequivalence testing in this patient population. Furthermore, AST has expressed caution regarding the currently unquantified risk that may be associated with switching immunosuppressive agents under uncontrolled circumstances and the need for clear labeling and patient education regarding any switch to or between generic formulations [6]. The KDIGO guidelines comment that because head-to-head data comparing efficacy and toxicity are generally not available for most generics, caution should therefore be exercised in choosing a generic formulation for use in kidney transplant recipients, and that ideally, a generic formulation should be used only after its safety and efficacy have been established in kidney transplant patients [3].

A recent study explored the effectiveness and safety of converting transplant patients from branded Prograf to generic TAC [7]. The single-center, retrospective, nonrandomized study reported that renal transplant patients experienced an average drop in the trough concentrations of TAC by 11.9% or 0.87 ng/mL [7]. Despite the drop in TAC concentrations, study investigators concluded that the conversion to generic TAC appears to be safe when coupled with “vigilant therapeutic drug monitoring” [7]. In response to these findings, as well as other recent case reports [8, 9], the transplant community continues to heighten awareness with respect to additional concerns for use of generic immunosuppressants, including the lack of research on patient and provider perspectives.

In one qualitative study in Australia, designed to obtain consumers' perspective on generic medicine use in general, patients reported a considerable mistrust of generic products [10]. This finding was confirmed in a recent survey of kidney transplant patients [11]. Although the findings from the Australian study are not directly related to a transplant population or immunosuppressants, the study also determined that substitution of a wide array of generic products and variability in their packaging added to consumers' overall concern, resulting in poor compliance [10]. Hesitation regarding the interchangeability of branded and generic therapies has been well documented throughout the literature for chronic diseases such as hypertension and congestive heart failure [12–14]; however, no studies have evaluated the impact of the growing availability of generic immunosuppressive agents and the consequences that may result for transplant coordinators and transplant patients. 

Preliminary reports suggest multiple switching among generic immunosuppressant products is occurring in practice and often without the knowledge of the provider [15]. Furthermore, patient confusion may be related to switches across multiple generic immunosuppression therapies [16], and provider workloads may increase due to recommended “vigilant therapeutic drug monitoring” upon switching a patient to a generic immunosuppressant [7]. Therefore, this study sought to examine transplant coordinator perspectives on their workload, patient confusion, patient education initiatives, and coordinators' perception, as to whether poor patient outcomes may have resulted from the multiple generic MMF and TAC therapies and the increasing complexity of immunosuppressants on the market.

## 2. Methods

An observational, cross-sectional, multicenter study was conducted via web-based survey. From August 2010 to September 2010, email invitations with a link to an online survey (see Appendix Supplementary Material available online at http://dx.doi.org/10.1155/2013/897434) and a description of the study were sent to transplant coordinators from a list of 200 practice managers within 106 transplant centers. The list of practice managers was based on convenience sampling and determined *a priori* to survey development. This study was designed to be a one-time questionnaire to view people's perception of workload over time. As such, the questionnaire used is not intended to be a validated instrument.

Transplant coordinators who were able to read English and complete an electronic web-based survey were included. Additionally, transplant coordinators must have provided informed consent at survey launch before initiation of survey questions. A screening question within the web-based survey was used to exclude transplant coordinators who did not participate directly in the management of outpatient immunosuppression therapy. Three followup emails were sent as reminders. No honorarium was provided for survey completion. Further, because this study was designed based on convenience sampling, no hypotheses were defined, and no power or sample size calculations were made. Upon completion of the survey, data was entered into a database and analyzed using descriptive statistics.

Liberty IRB (an independent central institutional review board) approved this study. Immunosuppression drug therapy selections were made by the clinician under conditions of routine care and were not impacted by the study.

## 3. Results

### 3.1. General and Demographic Data

Forty-six of the 106 transplant centers contacted (43%) completed or partially completed the online survey. Usable information was obtained from 28 transplant centers, which corresponded to 34 transplant coordinators. Transplant centers were primarily located in the south, midwest, and western regions of the United States (US), and more than half the centers performed more than 100 transplants annually (Table 1). The majority of transplant coordinators who responded to the survey were registered nurses, and 68% had more than five years of experience working with transplant patients.

### 3.2. Patient Confusion

**Figure 1:**
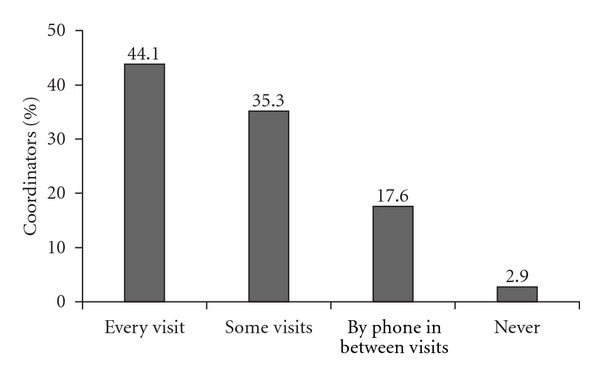
Frequency of patient questions regarding immunosuppressant therapy.

Transplant coordinators reported increased patient confusion over the past year for patients-receiving generic MMF (50%) or generic TAC (40%). Forty-four percent of the transplant coordinators reported that patients ask questions regarding their immunosuppressant therapy at every visit (Figure 1), and 18% reported that patients request information between visits via phone (Figure 1). Additionally, transplant coordinators reported that they answer at least three calls per day from patients (44%) and caregivers (20%) who have confusion regarding their immunosuppression therapy.

**Figure 2:**
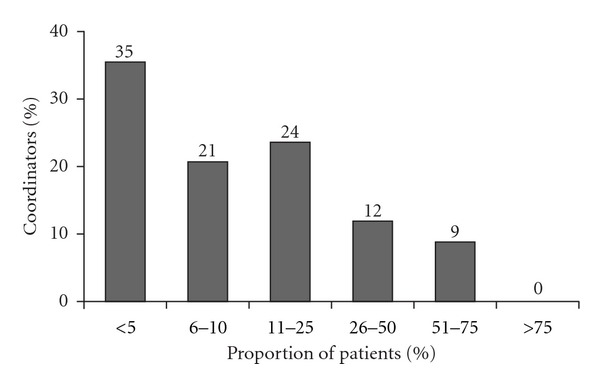
Proportion of patients confused about immunosuppressant therapy.

The surveyed sample of transplant coordinators also indicated that three out of every four patients (76.5%) are *often confused* or *sometimes confused* about their immunosuppression therapy. The remaining ones out of every four patients was reported to be *rarely confused* about their immunosuppression. Furthermore, when asked what proportion of their transplant patient population is at least *somewhat confused* about their immunosuppression therapy, 45% of the coordinators sampled reported that the proportion was greater than 10% (Figure 2). 

**Figure 3:**
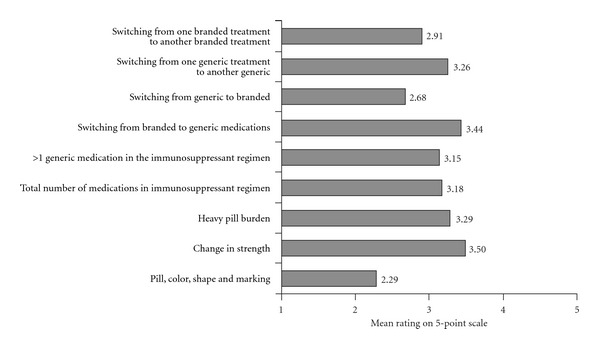
Immunosuppressant-related factors contributing to patient confusion.

Transplant coordinators indicated that *change in strength*, *switching from branded to generic medications*, *heavy pill burden*, and *switching from one generic to another generic* were the top four reasons for patient confusion regarding their immunosuppression therapy. Transplant coordinators ranked the factors contributing to patient confusion on a 5-point scale (1 = not a contributor; 5 = main contributor). Average scale ratings of transplant coordinator responses for the immunosuppressant therapy-related factors contributing to patient confusion can be located in Figure 3.

### 3.3. Transplant Coordinator Workload

**Figure 4:**
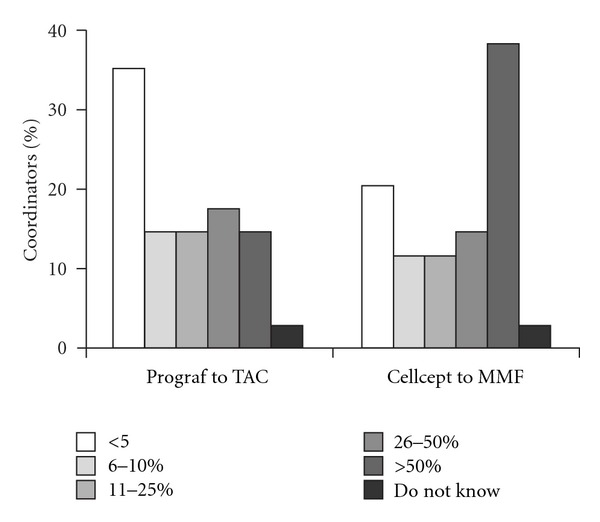
Proportion of patients switched to generic immunosuppression therapy. TAC, tacrolimus, MMF, and mycophenolate mofetil.

The majority of transplant coordinators who responded to the survey indicated an increase in workload since the introduction of generic MMF (62%) and TAC (65%) products. Fifteen percent of transplant coordinators reported that more than half of transplant recipients had switched from branded to generic TAC, and 38% reported more than half of recipients switched from branded to generic MMF (Figure 4).

Nearly one-third (29%) of transplant coordinators reportedly spend greater than 5 hours per week on the phone with patients discussing immunosuppressant therapy issues (Table 2). Similarly, more than one-quarter (27%) spend 2 hours per week on the phone with pharmacies, and 32% reportedly spend 1 hour weekly with other healthcare providers regarding immunosuppressant therapy issues. With respect to clinic visits, transplant coordinators estimated that the average duration of a patient clinic visit is from 30 minutes to 1 hour during the first year posttransplant; 62% of coordinators reported that more than 25% of the total clinic visit time posttransplant is spent on immunosuppressant education. The majority of transplant coordinators reportedly work in a center with a formal patient education plan (85%) and staff training on education programs (77%). Nearly 80% of transplant coordinators reported that nurses are primarily responsible for staffing patient education programs, and the same percentage reported that patient education initiatives have increased in the past year and are continuing to increase in importance. Additionally, in the past 1, 3, and 5 years, the majority of transplant coordinators (68%, 71%, and 62%, respectively) reported increased time spent on educating the patient about immunosuppressant therapy.

After *importance of adherence (taking medications daily) *and *side effects*, the *total number of medications*, *heavy pill burden*, and *change in pill strength* were rated the factors contributing most to the amount of education time required to inform a transplant recipient about his/her immunosuppressant therapy. These findings are supported by previous literature documenting the importance of educating transplant patients on the importance of adherence as a strategy for preventing adverse consequences [17].

Transplant coordinators were also surveyed on their knowledge of current availability of branded and generic immunosuppressant therapies. Three out of four transplant coordinators (76.5%) correctly identified which branded immunosuppressant therapy agents became generic over the past year (Cellcept and Prograf). The remaining correspondents (23.5%) reported knowing that there were generic products for Myfortic and Rapamune, although these agents are only available as branded products. Transplant coordinator responses were varied regarding the number of generics available for MMF and TAC. Transplant coordinator confusion, with respect to the availability of generic immunosuppressant agents, was further demonstrated by the fact that 38% of coordinators correctly identified how many different generic manufacturers of MMF were currently available, and 9% correctly identified the number of TAC generic manufacturers (at the time of survey completion).

### 3.4. Interruptions in Immunosuppressant Therapy

**Figure 5:**
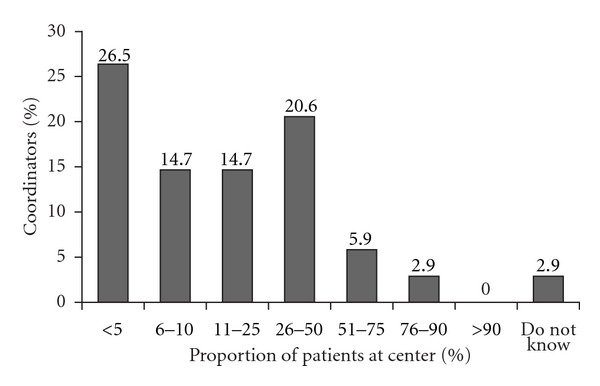
Transplant coordinator-reported proportion of transplant recipients experiencing adverse events during maintenance immunosuppression therapy.

*Patient out-of-pocket amount* and *reimbursement price *were reported to be the highest-ranking factors in terms of significance in the decision to substitute a generic for a branded immunosuppressant. Only half of the transplant coordinators reported that their transplant team had reviewed efficacy studies for generic agents since their introduction, and of those who had, 65% answered that the studies influenced prescribing patterns. For TAC and MMF, transplant coordinators reported *acute rejection rates*, *rate of graft loss*, and* poor patient adherence* to be the three most likely events to occur due to the availability of multiple generic immunosuppressants. (Note that each of these factors was rated 3 or higher on a 5-point scale, with “5” being very likely. The one exception was *poor patient adherence* for generic MMF, which received a rating of 2.97.) Respondents were also asked how many transplant patients at their center experienced adverse events during maintenance immunosuppression therapy: 41% of coordinators reported that 10% or fewer patients experienced adverse events (Figure 5).

## 4. Discussion

Posttransplant immunosuppression therapy regimens often contain an antimetabolite and a calcineurin inhibitor, such as MMF and TAC, respectively. With the recent introduction of several generic MMF and TAC options, concerns have been raised by patients and providers regarding the use of generics for maintenance immunosuppression therapy. The issues related to generic substitution can be compounded by the impact of multiple switches between generic formulations arising due to insurance coverage arrangements and availability at any given time at the pharmacy, beyond the control of the transplant center or the patient. Thus, this study sought to examine whether transplant coordinators feel the availability of multiple generic immunosuppressant therapies is associated with increased patient confusion and coordinator workload. The 34 coordinators surveyed, who represented a total of 28 centers, indicated that transplant recipient confusion and transplant coordinator workloads may increase due to the availability of multiple generic immunosuppressant therapies. These data are supported by previous findings that have linked increases in patient confusion as a result of generic substitution to decreased patient adherence and increased patient anxiety [14].

Although not the focus of this study, transplant coordinators also provided qualitative responses to some survey questions, in which several respondents stressed the increased workload, and time spent addressing patient concerns is primarily related to additional time spent educating patients, additional laboratory monitoring, and followup evaluations. This finding is likely attributable to guideline-suggested therapeutic drug monitoring and followup visits upon changing a patient's calcineurin inhibitor regimen [3] and supported by recently published data regarding the direct comparison of branded versus generic TAC [7]. Despite increasing efforts to implement therapeutic drug monitoring for MMF into clinical practice [18], MMF concentrations are not routinely monitored [3]. Studies have demonstrated a potential link between mycophenolic acid concentrations, the risk of allograft rejection [18–20], and a high degree of intrapatient variability of mycophenolic acid pharmacokinetics. The introduction of several generic MMF products may therefore further decrease the predictability of a patient's mycophenolic acid concentrations and warrant routine therapeutic drug monitoring, adding even more to the workload of coordinators and the amount of healthcare resources consumed in these patients. Safe and effective conversion to generic immunosuppression therapies may require additional monitoring and followup efforts [7], resulting in the consumption of greater healthcare resources. Additionally, it has been estimated that the cost of a therapeutic drug and allograft monitoring may cost more than $750 per visit [15]. Lastly, the burden on patients in terms of blood sampling and inconvenience would be high, particularly for generic formulations of MMF for which measurement of abbreviated area under the curve (AUC) would be required for accurate assessment of exposure, with possible consequences for patient compliance. Ultimately, the perceived cost savings associated with generic immunosuppressants may be neutralized or, at best, offset by the additional testing and procedures required upon product switching. 

Transplant coordinators also provided comments regarding the coverage-related challenges associated with providing branded immunosuppression therapies to patients. Transplant coordinators are often responsible for obtaining prior authorization from insurance companies for branded immunosuppression therapy, thus increasing their workload. Switching to generic immunosuppression therapy would eliminate the added time of obtaining prior authorization and may result in reduced workload for transplant coordinators. One of the limitations of this study is that we did not ask any questions around this topic or around the number of patients that cannot pay for the branded medications due to insurance coverage, and so forth.

We recognize that there are other limitations of the study that merit consideration. First, it lacked the perspective of the patient and relied upon transplant coordinators to generalize and estimate their perceptions of their individual patient populations. Thus, the transplant coordinators inferred the level of confusion about the immunosuppression regimen based on their experience, and, specifically, the number of phone calls they received with enquiries from patients or caregivers but no patient-reported data on confusion was collected. Second, fewer than half the transplant centers that were contacted responded, with a consequent potential for the introduction of bias in the results. Third, the study did not include any assessment of how patient demographics, and, for example, level of education may have influenced the degree of patient confusion. Fourth, the scope of the survey did not permit a detailed examination of transplant coordinators' impression of the incidence of adverse events in patients receiving specific branded or generic immunosuppressive agents. Lastly, while respondents were asked what events they considered most likely to occur due to the availability of multiple generic preparations of TAC and MMF, their feedback was opinion based and not derived from events occurring at their center. 

 The consensus among the transplant community is that generic immunotherapy can provide a safe alternative to branded drugs, but certain precautions must be followed. Patients and transplant coordinators must be aware of the switch to generic, the same generic drug should be administered continuously to receive consistent benefit, and stringent therapeutic drug monitoring must be administered during the initial switch phase [21]. The impact on transplant coordinators has not previously been assessed. Although no testing for statistical significance was performed, the key findings of this study are likely experienced by most, if not all transplant centers throughout the US. Increased awareness among transplant coordinators of the challenges brought about by multiple generic immunosuppressants may therefore be necessary to identify and mitigate patient care-related issues. Additional studies will also be necessary to address the primary limitation of this study and gain insights directly from patients regarding generic immunosuppression therapies. Furthermore, given the reimbursement and access challenges that already face this patient population [22–25], additional research is warranted to identify how minimizing patient confusion and coordinator workload may lead to improved outcomes. 

## 5. Conclusion

Transplant centers must evaluate the value of multiple generic immunosuppressive therapies in the context of challenges to transplant patients and transplant coordinators. Increases in transplant coordinator workload, patient confusion, and potential interruptions in therapy all introduce additional risks to a population that already takes multiple medications, where nonadherence could be detrimental to graft outcomes. Furthermore, the perceived cost savings associated with generic therapies need to be balanced with necessary therapeutic drug monitoring, and the additional health care resources needed to safely and effectively transition patients to generic immunosuppressants. Ultimately, efforts to optimize therapy and remove some of the risk for poor outcomes should become a priority for all stakeholders involved in delivery and access to posttransplant care.

## Supplementary Material

The Myfortic Transplant Coordinator Survey was a four-part electronic web-based survey which was sent to transplant coordinators in the US. The first section requested information on the respondent's demographics, including their experience and role, and the number of transplants performed at their center. The second section comprised seven questions about patient confusion regarding immunosuppressive medication, such as how frequently patients asked questions about their therapy, the number of telephone calls the respondent answered per day relating to immunosuppressive therapy, and their assessment of patient confusion levels. The third section of the survey focused on the impact on the respondent's workload, in terms of time required to answer questions about immunosuppressive medication, whether this workload had changed in recent years, what factors influenced the amount of patient education required, and whether introduction of generic immunosuppressant medications had affected workload. The final section comprised nine questions concerning interruptions or changes to immunosuppressive therapy, and the use of generic preparations. Questions primarily offered multiple-choice answers, with a small number requesting free text responses.

## Figures and Tables

**Table 1:** Transplant coordinator demographics.

Characteristic	Transplant coordinators, *n* (%) (*N* = 34)
US region	
North	2 (5.9)
Midwest	6 (17.6)
West	7 (20.6)
South	19 (55.9)
Number of solid organ transplants performed annually at transplant center	
1–25	2 (5.9)
26–50	4 (11.8)
51–75	1 (2.9)
76–100	9 (26.5)
101–200	10 (29.4)
>200	8 (23.5)
Role/title of transplant coordinator	
Registered nurse	28 (82.4)
Certified registered nurse practitioner	4 (11.8)
Physician assistant	1 (2.9)
Other	1 (2.9)
Years spent working with transplant patients	
<1 year	1 (2.9)
2-3 years	3(8.8)
4-5 years	7 (20.6)
>5 years	23 (67.6)

**Table 2:** Coordinator time spent on telephone regarding immunosuppressant therapy issues.

	Number of hours per week						
Hour(s)	0	0.5	1	1.5	2	2–5	>5

	% transplant coordinators						

Patients	2.9	8.8	14.7	2.9	17.6	23.5	29.4
Caregivers	5.9	26.5	14.7	8.8	17.6	14.7	11.8
Pharmacies	0.0	17.6	5.9	11.8	26.5	20.6	17.6
Other healthcare providers	14.7	11.8	32.4	14.7	8.8	11.8	5.9
Insurers/health plans	17.6	8.8	11.8	17.6	17.6	14.7	11.8
