# Supplementary material for: Transplant Coordinators' Perceived Impact of Availability of Multiple Generic Immunosuppression Therapies on Patients, Workload, and Posttransplant Maintenance Therapy
Source: J Transplant. 2013 Jan 8;2013:897434. doi: 10.1155/2013/897434 (PMC3556842; doi:10.1155/2013/897434)
Supplement: Supplementary file 1 — The Myfortic Transplant Coordinator Survey was a four-part electronic web-based survey which was sent to transplant coordinators in the US. The first section requested information on the respondent's demographics, including their experience and role, and the number of transplants performed at their center. The second section comprised seven questions about patient confusion regarding immunosuppressive medication, such as how frequently patients asked questions about their therapy, the number of telephone calls the respondent answered per day relating to immunosuppressive therapy, and their assessment of patient confusion levels. The third section of the survey focused on the impact on the respondent's workload, in terms of time required to answer questions about immunosuppressive medication, whether this workload had changed in recent years, what factors influenced the amount of patient education required, and whether introduction of generic immunosuppressant medications had affected workload. The final section comprised nine questions concerning interruptions or changes to immunosuppressive therapy, and the use of generic preparations. Questions primarily offered multiple-choice answers, with a small number requesting free text responses. [file 897434.f1.pdf]

Xcenda
AmerisourceBergen Specialty Group

Myfortic Transplant Coordinator Survey: Defining the perceptions of generic mycophenolate mofetil

July 28, 2010

Prepared for:
Steve Arcona, PhD
Director, Outcomes Research
Novartis Pharmaceuticals Corporation

proving**value.**maximizing**access.**™

Please direct comments and questions to:

Jay Jackson, PharmD, MPH
Vice President, Medical Services
Xcenda
4114 Woodlands Parkway, Suite 500
Palm Harbor, FL 34685
Phone: 727-771-4133
Fax: 727-771-4145
Email: jay.jackson@xcenda.com

Amanda Gilmore, PhD, MPH
Director, Medical Services
Xcenda
4114 Woodlands Parkway, Suite 500
Palm Harbor, FL 34685
Phone: 504-267-0442
Fax: 727-771-4145
Email: amanda.gilmore@xcenda.com

**Coordinator Demographics**	
1.	How many solid organ transplants are performed annually at your transplant center?<br>□ 10-25 □ 26-50 □ 51-75 □ 76-100 □ 101-200 □ >200
2.<br>(screening question)	Do you participate directly in the management of transplant recipients' outpatient immunosuppression?<br>□ Yes □ No
3.	How many years have you worked with transplant patients?<br>□ ≤1 year □ 2-3 years □ 4-5 years □ >5 years
4.	Which of the following describes your primary job function<br>□ RN □ CRNP □ PA □ MD □ PharmD □ RPh □ DO □ Other
**Understanding Patient Confusion**	
5.	How often do patients in your practice ask questions regarding their immunosuppressant therapy?<br>□ Every visit □ Some visits □ By phone in between visits □ Never
6.	In general, rate your transplant patient's level of confusion regarding their immunosuppression regimen?<br>1—Completely confused 5—No confusion
7.	How many phone calls do you answer per day from patients who have confusion regarding their immunosuppression therapy?<br>□ 0 □ 1 □ 2 □ 3 □ 4 □ 5 □ >5
8.	How many phone calls do you answer per day from caregivers who have confusion regarding a transplant recipients' immunosuppression therapy?<br>□ 0 □ 1 □ 2 □ 3 □ 4 □ 5 □ >5
9.	What proportion of your total transplant patient population is at least somewhat confused about their immunosuppression?<br>□ <5% □ 6-10% □ 11-25% □ 26-50% □ 51-75% □ 76-90% □ >90%

10.	Rate the below factors based on how much each contributes to a transplant recipient's confusion regarding their immunosuppressant therapy.<br>1—Is not a contributor to patient confusion 5—Is a main contributor to patient confusion<br><br>□ Pill Color, Shape, Marking<br>□ Change in strength<br>□ Heavy pill burden<br>□ Total number of medications in immunosuppressant regimen<br>□ >1 generic medication in the immunosuppressant regimen<br>□ Switching from branded to generic medications<br>□ Switching from generic to branded<br>□ Switching from one generic treatment to another generic<br>□ Switching from one branded treatment to another branded treatment
11.	Are there certain patient factors that tend to increase the level of confusion surrounding the immunosuppressant regimen?<br>□ Yes □ No<br><br>If yes, please describe (free text).<br>____<br>____
**Impact on Coordinator Workload**	
12.	Estimate how many hours per week you spend on the phone with the following groups discussing immunosuppressant therapy-related issues?<br>0 hours ½ hour 1 hour 1 ½ hours 2 hours 2 – 5 hours >5 hours<br><br>□ Patients □ Caregivers □ Pharmacies □ Other HCPs □ Insurers/Health Plans
13.	On average, how long is a recipient's clinic visit time in the first year following transplantation?<br>□ <30 minutes □ 30 minutes to 1 hour □ >1 hour to 1.5 hours □ >1.5 hours<br><br>On average, how much of the total post-transplant clinic visit time is spent on educating the patient about his/her immunosuppressant therapy?<br>□ <5% □ 6-10% □ 11-25% □ 26-50% □ 51-75% □ 76-90% □ >90%
14.	Which of the following best describes your practice in terms of patient education initiatives? (Pick one.)<br>□ Patient education initiatives have remained the same over the past year.<br>□ Patient education initiatives have increased over the past year and are gaining more importance.<br>□ Patient education initiatives have increased over the past year but are becoming less important.<br>□ Patient education initiatives have decreased over the past year but are gaining more importance.

	□ Patient education initiatives have decreased over the past year and are becoming less important.
15.	Does your transplant center have a standard operating procedure regarding patient education?<br>□ Yes □ No □ Do not know
16.	Is the staff in your transplant center trained on patient education programs?<br>□ Yes □ No □ Do not know
17.	In your transplant center, which of the following staff members are responsible for staffing patient education programs? (Select all that apply.)<br>□ Physician □ Nurse □ Pharmacist □ Combination/Integrated
18.	In general, how has the amount of time spent on educating the patient about his/her immunosuppressant therapy changed? in the past year? 3 years? 5 years?<br>□ Increased □ Decreased □ Remained the same □ Do not know
19.	Rate the below factors based on how much each contributes to the amount of education time required to adequately inform a transplant recipient about his/her immunosuppressant therapy.<br><br>1—No education time required 5—Is a main contributor to the amount of time spent educating the patient<br><br>□ Pill Color, Shape, Marking<br>□ Change in pill strength<br>□ Heavy pill burden<br>□ Total number of medications in immunosuppressant regimen<br>□ >1 generic medication in the immunosuppressant regimen<br>□ Medication side effects<br>□ Importance of adherence (taking medications daily)<br>□ Switching from branded to generic medication<br>□ Switching from generic to branded medication<br>□ Switching from one generic treatment to another generic<br>□ Switching from one branded treatment to another branded treatment
20.<br>(not mandatory)	What recent changes in educating a patient about immunosuppressant therapy have you observed? (Free text)
21.	Over the past year, generic products for which of the following immunosuppressants have become available? (Select all that apply.)<br>□ CellCept® □ Myfortic® □ Prograf® □ Rapamune®

22.	How many generic versions of mycophenolate mofetil are currently available?<br>□ 0 □ 1 □ 2 □ 3 □ 4 □ 5 □ >5
23.	How many generic versions of tacrolimus are currently available?<br>□ 0 □ 1 □ 2 □ 3 □ 4 □ 5 □ >5
24.	Overall, what proportion of transplant recipients in your center has switched from branded Prograf® to a generic tacrolimus product?<br>□ <5% □ 6-10% □ 11-25% □ 26-50% □ 51-75% □ 76-90% □ >90% □ Do not know<br><br>Overall, what proportion of transplant recipients in your center has switched from branded Cellcept® to a generic MMF product?<br>□ <5% □ 6-10% □ 11-25% □ 26-50% □ 51-75% □ 76-90% □ >90% □ Do not know
25.	Which of the following best describes your workload since the introduction of generic immunosuppressants? (Pick one.)<br><br>□ My workload has increased as a result of new generic immunosuppressants.<br>□ My workload has decreased as a result of new generic immunosuppressants.<br>□ My workload has remained the same with new generic immunosuppressants.<br>□ I don't know.<br><br>Which of the following best describes your workload since the introduction of multiple generic MMF products? (Pick one.)<br><br>□ My workload has increased as a result of new generic immunosuppressants.<br>□ My workload has decreased as a result of new generic immunosuppressants.<br>□ My workload has remained the same with new generic immunosuppressants.<br>□ I don't know.<br><br>Which of the following best describes your workload since the introduction of multiple generic tacrolimus products? (Pick one.)<br><br>□ My workload has increased as a result of new generic immunosuppressants.<br>□ My workload has decreased as a result of new generic immunosuppressants.<br>□ My workload has remained the same with new generic immunosuppressants.<br>□ I don't know.
26.	Describe how your workload has changed since the introduction of generic immunosuppressant medications?
**Interruptions in Immunosuppressant Therapy**	
27.	Please rate each of the following factors based on its level of significance in the decision to substitute a generic immunosuppressant for a branded immunosuppressant.<br>1—Not significant 5—Very significant

	□ Patient preference □ Adverse reactions □ Reimbursement price<br>□ Patient out-of-pocket amount (i.e., copay) □ Physician autonomy □ Monitoring parameters □ Efficacy Studies □ Economic studies / models □ Therapeutic Index
28.	Since the introduction of generic immunosuppressants, has your team reviewed any efficacy studies for generic agents in transplant patients?<br>□ Yes □ No □ Do not know<br><br>If yes, did those studies influence prescribing patterns for immunosuppressant therapy?<br>□ Yes □ No □ Do not know<br><br>If no, would your practice review efficacy studies for generic immunosuppressant therapy?<br>□ Yes □ No □ Do not know
29.	There are currently 9 generic MMF products on the market. Rate each of the below factors based on your perception of their likelihood of occurring, due to the availability of multiple generic products.<br>1—Not Likely 5—Very Likely<br><br>□ Poor patient adherence □ Missed doses □ Acute rejection rate □ Rate of graft loss<br>□ Mortality rate<br><br>There are currently several generic tacrolimus products on the market. Rate each of the below factors based on your perception of their likelihood of occurring, due to the availability of multiple generic products.<br>1—Not Likely 5—Very Likely<br><br>□ Poor patient adherence □ Missed doses □ Acute rejection rate □ Rate of graft loss<br>□ Mortality rate
30.	Transplant patients in our center are encouraged to bring their medications to every office visit for adherence monitoring and patient education.<br>□ Yes □ No □ Do not know
31.	What percentage of patients in your practice actually bring their immunosuppressant medications with them during office visits?<br>□ <5% □ 6-10% □ 11-25% □ 26-50% □ 51-75% □ 76-90% □ >90% □ Do not know
32.	Check all of the following that apply to your transplant center. Patients are instructed to call the office if any of the following occur:<br>□ Missed dose □ Pharmacy changes their prescription to a different generic<br>□ Pharmacy changes their prescription from generic to brand<br>□ Pharmacy changes their prescription from brand to generic<br>□ Experience an adverse event □ Another doctor prescribes medicine<br>□ Patient desires to use OTC medicine

33.	How many transplant patients at your center experience adverse events during maintenance immunosuppression therapy?<br><br>□ <5% □ 6-10% □ 11-25% □ 26-50% □ 51-75% □ 76-90% □ >90% □ Do not know
34.	Check all of the following that apply to your patients. Over the past year, have you observed an increase in any of the following adverse events in patients who are taking generic MMF.<br>□ Anemia □ Gastritis □ GI (Diarrhea, constipation, nausea, dyspepsia) □ GI bleed<br>□ Headache □ Leukopenia □ Neutropenia □ Rash □ Tremor □ Graft Loss □ Acute Rejection □Increased Patient Confusion
35.	Check all of the following that apply to your patients. Over the past year, have you observed an increase in any of the following adverse events in patients who are taking generic tacrolimus.<br>□ Anemia □ Gastritis □ GI (Diarrhea, constipation, nausea, dyspepsia) □ GI bleed<br>□ Headache □ Leukopenia □ Neutropenia □ Rash □ Tremor □ Graft Loss □ Acute Rejection □Increased Patient Confusion
